# Probiotic‐Based Approaches for Sustainable Control of Infectious Risk in Mass Transport: Current Data and Future Perspectives

**DOI:** 10.1111/1751-7915.70177

**Published:** 2025-06-14

**Authors:** Irene Soffritti, Maria D'Accolti, Francesca Bini, Eleonora Mazziga, Antonella Volta, Matteo Bisi, Sante Mazzacane, Elisabetta Caselli

**Affiliations:** ^1^ Section of Microbiology, Department of Environmental and Prevention Sciences University of Ferrara Ferrara Italy; ^2^ CIAS Research Centre, Tekne‐Hub, University of Ferrara Ferrara Italy

**Keywords:** built environment, mass transport, microbiome, probiotics

## Abstract

The built environments of high‐traffic areas can play a significant role in the transmission of microorganisms and associated infections, sometimes favouring the selection of multidrug‐resistant (MDR) organisms due to the excessive use of conventional disinfectants. Probiotic‐based sanitation (PBS) was suggested as a novel alternative approach to control the infectious risk in crowded community environments due to its effectiveness in reducing fungal, bacterial, and viral pathogens in sanitary settings. PBS may thus trigger a paradigm shift from chemical to biological strategies in cleaning environments with high human occupancy, offering an ecological and economically sustainable alternative to conventional chemical disinfection. Providing robust data supporting the results reported so far, it has the potential to optimise bioburden control and infection prevention in mass transportation spaces. This review brings together existing research on PBS in mass transportation areas, pinpoints areas of lack of information, and explores its potential future uses, including the creation of probiotic‐based materials for sustainable biocontrol in high‐traffic areas.

## Graphical abstract

High‐traffic areas, such as mass transportation environments, can have a significant impact on infection transmission, and chemical disinfection may favour the selection of drug‐resistant microbes. Probiotic‐based approaches have the potential to be a novel and ecologically sustainable option for controlling bioburden and preventing infections.

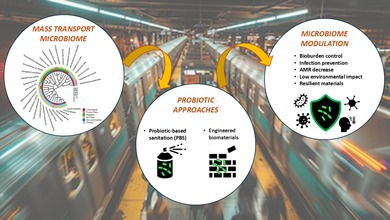

## 1 Introduction

The urban environment currently hosts around 55% of the world's population (United Nations 2018; Ritchie et al. 2020), while the majority lived in rural areas or small villages until the last century. Consequently, most modern humans spend most of their lives indoors, often in highly populated built environments (BEs) such as workplaces, educational institutes, healthcare facilities, and public transportation. These high‐traffic areas become thus rapidly colonised by microbes spread by human occupants, evolving an indoor microbial population mostly derived from humans. By contrast, unrestricted environments are mostly populated by microorganisms derived from the outdoor environment, which are associated with higher biodiversity and less pathogenic potential (Young et al. 2023).

Similarly to what is recognised for living organisms, including human beings, BEs are currently recognised as super‐ecosystems, since they develop their own microbiome, whose features depend essentially on how much the BE is confined and controlled through the use of antimicrobials. Specifically, the microbiome of more restricted BEs has been recognised to have mostly an anthropic origin, being composed of bacteria, viruses, and fungi spread by the human beings occupying those BEs (Zilber‐Rosenberg and Rosenberg 2008; Berg et al. 2020). Consequently, the microbiome of restricted BEs shows less biodiversity, in terms of species richness, and more antimicrobial resistance (AMR), compared to the microbiome of unrestricted environments (Mahnert et al. 2019). Microbes can persist for prolonged times in BE areas, spreading in both surface and air and potentially being transmitted to other humans within the BE (Smith et al. 1996; Kramer et al. 2006; Otter and French 2009).

In urban settings, urban transit systems, including subways, trains, and buses, serve as a daily point of contact for billions of city residents. Urban travellers move through these systems, spreading their own microorganisms and, in turn, coming into contact with BE microorganisms via contact with highly touched surfaces and inhalation of shared air (Ly et al. 2024). In these high‐traffic BEs, the transmission of pathogens can potentially and rapidly impact the health of a high number of people; hence, BE sanitation is crucial to control the infectious risk in those areas and preserve the health of human occupants.

To achieve this goal, conventional disinfection has been the most commonly used method so far. However, disinfectants' usage has some general concerns that are recognised to be associated with them, such as a significant impact on earth and water pollution, limited persistence of action, and the possibility of induction of AMR (Nabi et al. 2020; Zhang et al. 2020). The most frequently used chemical disinfectants include quaternary ammonium compounds (QAC), chlorine and chlorine derivatives, alcohols and phenols (CDC 2008). All of them have some key drawbacks that can directly or indirectly affect human health. First, disinfectants have a temporary effect that lasts on surfaces for minutes to 1–2 h after application, making them ineffective in preventing recontamination, which occurs continuously in high‐traffic BEs (D'Accolti, Soffritti, Bonfante, et al. 2021). In addition, an increasing number of studies have recognised chemical disinfectants as major contributors to the onset of resistance to disinfectants themselves and cross‐resistance to antibiotics (Kampf 2018). Besides chemical disinfection, other bioburden control measures include the use of UV‐C, fumigation, antimicrobial surfaces, and plasma air sterilisation (Ly et al. 2024). The evaluation and optimisation of these strategies is ongoing. Application costs, variable effectiveness depending on material type, and incompatibility with human presence are the main limitations so far.

Among the recent innovative approaches developed to address the urgent need for cost‐effective, safe, and environmentally friendly sanitation solutions, the probiotic‐based sanitation (PBS) has emerged as an interesting and effective approach. This review summarises some of the comparative studies that were performed by using PBS as a substitute for chemical disinfection in mass transportation areas, reporting the current data and highlighting those that are still missing. The use of probiotics in innovative materials for biocontrol is also presented.

### 1.1 The Microbiome of Built Environments (BEs)

The BE microbiome is a dynamic and complex ecosystem, influenced by continuous interactions among microorganisms, the environment, and building occupants (Dai et al. 2017; Mahnert et al. 2019). Research data show significant variability in the structure, abundance, and diversity of the BE microbiome across different indoor spaces (Adams et al. 2015; Shin et al. 2015; Bragoszewska and Biedroń 2018). In addition, the composition of the BE microbiome also depends on factors such as geography, seasons, and human activities (Rai et al. 2021). Human occupants are the main contributors to the BE microbiome, spreading their microbes into indoor spaces, and areas with greater traffic have a higher abundance of microbes of human origin. In addition, human movements can stir and resuspend settled particles, bringing them into the BE air (Adams et al. 2016).

According to this, indoor microbiomes can be “healthy” if they contain beneficial commensal microbes introduced by healthy individuals and/or pets and plants. According to the Human Microbiome Project, the principal sources of human microorganisms include the oral and nasal cavities, vagina, intestines, and skin, which are key research subjects studied by the scientific community (Turnbaugh et al. 2007). Among them, the oral and nasal tracts are significant interfaces between humans and the environment. The microbial elements they carry can spread through aerosols and increase the indoor air microbial burden by approximately 10^4^–10^6^ bacteria per m^3^ (Hewitt et al. 2012; Gaüzère et al. 2014). Similarly, the skin has the ability to spread approximately 15 × 10^6^ bacteria per hour (Kelley and Gilbert 2013). As a result, human contact creates a unique microbial signature on surfaces and the surroundings, disseminating prominent bacterial phyla such as *Firmicutes*, *Bacteroidetes*, and *Proteobacteria* (Wilkins et al. 2016). Despite numerous studies on the composition of various microbial communities found in different BE compartments and specific locations (such as offices, subways, hospitals, etc.), the definition of a “healthy” BE microbiota remains elusive (Dannemiller 2019). Overall, research on BE microbial communities reveals the presence of bacteria from four major phyla, including *Proteobacteria*, *Bacillota*, *Actinomycetota*, and *Bacteroidota*, along with less represented groups like *Aquificota*, *Chlamydiota*, and *Cyanobacteriota* (Zampolli et al. 2024).

*Protobacteria* (*Pseudomonadota* phylum), which includes a variety of Gram‐negative bacteria, is particularly prevalent. Among *α‐Proteobacteria*, *Methylobacterium*, *Sphingomonas*, *Bradyrhizobium*, *Neorhizobium*, and *Rhizobium* can commonly be found indoors (Hewitt et al. 2012; Kelley and Gilbert 2013; Adams et al. 2017; Merino et al. 2019; Rai et al. 2021; Cao et al. 2021). Additionally, *α‐Proteobacteria* such as *Bosea*, *Rhodobacter*, and *Brucella* have been frequently detected in BEs like offices, museums, and shopping centres (Wilkins et al. 2016; Gilbert and Stephens 2018); *Paracoccus* has been found in office and museum bioaerosols (Gaüzère et al. 2014; Adams et al. 2017), and *Brevundimonas* has been detected in subway and university BEs (Adams et al. 2017; Merino et al. 2019). Among *β‐Proteobacteria*, *Bordetella*, *Burkholderia*, and *Neisseria* are commonly found indoors and are associated with a high amount of human occupancy (Kelley and Gilbert 2013; Prussin and Marr 2015; Gilbert and Stephens 2018; Merino et al. 2019). Among *γ‐Proteobacteria*, genera often reported in the BE microbiome are *Acinetobacter* and *Pseudomonas*. Additionally, *Enterobacter* and *Escherichia* are commonly detected indoors, serving as indicators of faecal contamination (Leri and Khan 2023). The *Bacillota* phylum, primarily consisting of Gram‐positive bacteria, includes genera such as *Bacillus* and *Staphylococcus*, which are commonly found in indoor environments such as offices, museums, and gyms around the world (Zampolli et al. 2024). Among the *Actinomycetota* phylum, the most prevalent genera detected in the BE were *Corynebacterium*, *Mycobacterium*, *Propionibacterium*, *Streptomyces*, and *Rhodococcu*s (Zampolli et al. 2024). The *Bacteroidota* phylum, which includes Gram‐negative bacteria found also in the human gut and skin, was also detected. In particular, *Prevotella* and *Bacteroides* genera were frequently detected in household air and offices (Hewitt et al. 2012; Prussin and Marr 2015; Wilkins et al. 2016; Gilbert and Stephens 2018; Merino et al. 2019). These genera, which are part of the human microbiota, also serve as indicators of faecal contamination, and their abundance tends to increase with urbanisation (Browne et al. 2017; Rai et al. 2021).

Differently, BE microbiomes can be considered “unhealthy” when they contain a significant proportion of pathogenic microorganisms, which may be introduced by infected individuals and eventually selected due to the extensive use of antimicrobials (disinfectants and antimicrobial drugs). This kind of BE microbiome is especially observed in highly restricted and controlled BEs, such as hospitals. The microbiome there becomes a reservoir of multidrug‐resistant (MDR) microbes, which are causally related to the so‐called healthcare‐associated infections (HAI) (Pittet et al. 2000; Tacconelli et al. 2014; Li et al. 2021; Hu et al. 2022).

Besides sanitary BEs, the prolonged exposure to unhealthy BE microbiomes can have a significant impact on human health even in non‐sanitary BEs. In fact, compared to what is observed in natural rural environments, there was a reported increase in the risk of acquiring various diseases (Dai et al. 2017). BE surface and air microbiome, especially in conditioned‐air spaces, can actually become a reservoir for pathogens even in non‐sanitary settings. Consistently, prolonged exposure to poor air quality in BEs has been associated with the development of the “Sick Building Syndrome” (SBS), characterised by nonspecific symptoms such as headaches, eye and throat irritation, fatigue, nausea, and difficulty in concentrating (EPA 1991; Prussin and Marr 2015). SBS can be exacerbated by contaminants such as bacteria, fungal spores, and moulds that thrive in specific temperature and humidity conditions by spreading through ventilation systems (Joshi 2008). Compared to unrestricted environments, restricted ones generally have microbiomes with reduced biodiversity and increased AMR (Kang et al. 2018; Mahnert et al. 2019; Nowrotek et al. 2019). These conditions are typically observed in hospitals, but they can also be found wherever disinfectants and antimicrobials are routinely applied, exerting a constant selective pressure on the indoor microbiome. These environments include agricultural and animal husbandry settings (Chokshi et al. 2019), as well as domestic environments (Jovel et al. 2016; Li et al. 2018; Xu et al. 2018). Loss of biodiversity in the BE microbiome was associated with increased risks of allergies, asthma, and other chronic conditions (Flandroy et al. 2018). By contrast, early exposure to an environment rich in biodiversity appears to be crucial for health (Hanski et al. 2012). Findings suggest that up to 25% of variability in the human microbiome is attributed to environmental factors, rather than genetic background, emphasising the fundamental role of the surrounding environment in human health and disease conditions (Rothschild et al. 2018). Consistently, children growing in rural environments, which offer higher microbial biodiversity, have a reduced risk of respiratory inflammation compared to urban children (Havstad et al. 2011; Dominguez‐Bello et al. 2016).

As stated before, the microbiome of confined BEs mainly consists of bacteria that come from humans, typically including skin colonisers such as Gram‐positive *Staphylococcus* spp. and frequently exhibiting detectable levels of Gram‐negative *Enterobacteriaceae*, fungi, and viruses. The Home Microbiome Project studies showed a strong connection between household microbes and their residents, indicating the quick colonisation of indoor spaces by human‐associated microbiota (Lax et al. 2014; Li et al. 2021). Microbes can quickly colonise toilets, kitchens, and refrigerators, potentially leading to illnesses (Jeon et al. 2013). Dry indoor environments are especially resilient for *Staphylococcus* species, which could be responsible for the onset of diseases (Shan et al. 2020). It is worth noting that antibiotic‐resistant *Staphylococcus* strains have recently been reported even in community/domestic spaces, including methicillin‐resistant *Staphylococcus aureus* (MRSA), which was previously detected almost exclusively in hospitals (Cave et al. 2021; D'Accolti et al. 2023b). Also, MDR coagulase‐negative *Staphylococcus* species (such as *S. epidermidis* , *S. haemolyticus* , *S. hominis* and *S. saprophyticus* ), previously mainly detected in the hospital environment, can now be frequently found in non‐sanitary environments (Davis et al. 2012; Becker et al. 2014).

Besides Staphylococci, bacteria belonging to the Gram‐negative *Enterobacteriaceae* family, representing a prevalent part of the human gut microbiome, can often be detected in indoor spaces. These bacteria include MDR strains exhibiting resistance against beta‐lactams and carbapenems (Denisuik et al. 2013; Kelly et al. 2017).

Indoor environments are also home to fungi species such as *Cladosporium*, *Aspergillus*, and *Penicillium*. Indoor air pollution is caused by these microorganisms, which can remain on surfaces for extended periods and release spores, hyphal fragments, and mycotoxins (Nevalainen et al. 2015; Flannigan et al. 2016). Viruses can also be detected indoors, and virus sources include humans, pets, plants, ventilation systems, and dust (Prussin and Marr 2015). Even though the BE virome has yet to be fully explored, the SARS‐CoV‐2 pandemic highlighted the crucial role of indoor spaces in virus transmission, both through direct human contact and airborne routes (Cai et al. 2020; Dietz et al. 2020; Liu et al. 2020). Contaminated surfaces and fomites are also a way for viruses to spread, with many being able to persist on inanimate surfaces for days (Kampf et al. 2020). Enveloped viruses like SARS‐CoV‐2, influenza, and herpesviruses are also included in this group (Kramer et al. 2006; Dublineau et al. 2011). In sanitary settings, inanimate surfaces were found to hold almost all kinds of human viruses, which were linked to the onset of healthcare infections of viral origin, particularly in critically ill patients (Chow and Mermel 2017; Fragkou et al. 2021; Xiang et al. 2023).

### 1.2 Common Pathogens Associated With Mass Transportation

**FIGURE 1:**
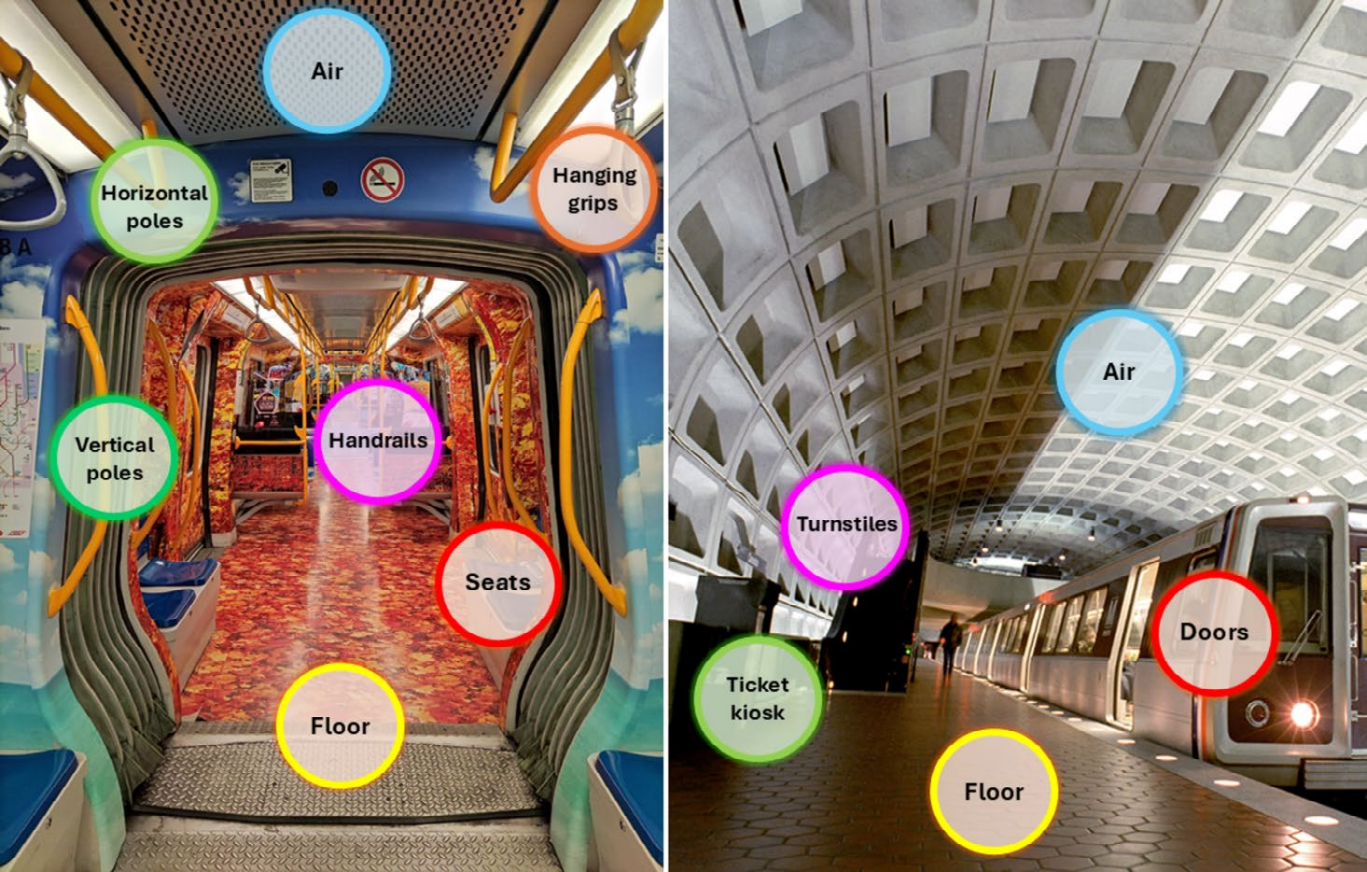
Frequently contaminated surfaces in the subway environment.

While travelling, we carry our microbes with us and spread them throughout the environment. Consequently, mass transportation systems are spaces where the flow and exchange of microbes between humans and transport BE occur continuously. These microbes are able to spread efficiently by either touching surfaces or inhaling aerosols found in passenger cabins, leading to a rapid impact on large populations (Hsu et al. 2016; Zhang et al. 2016). The majority of research has been currently focused on subways and trains among various transportation systems. Large cities' subways transport millions of passengers daily and represent a unique microbial ecosystem that is characterised by high density, diversity, and turnover of occupants, which enhances the flow of human microbes (Ly et al. 2024). Subway microbes can be transmitted to human occupants via direct human‐to‐human contact but also through indirect contact with frequently touched surfaces (i.e., handrails) and shared air in the subway spaces (Figure 1).

Subways usually have air conditioning systems using a high air flow rate and speed, which further promote the spread of physical (powders with different grain sizes), chemical (CO_2_ and volatile organic compounds, VOCs), and microbial contaminants (Wen et al. 2020). The transfer of human hand microbes from test subjects to objects was demonstrated by various studies (Hsu et al. 2016; Zhang et al. 2016), and the virus's high transmissibility in these environments was brought into focus by the SARS‐CoV‐2 pandemic (Chin et al. 2020; Marquès and Domingo 2021). Consistent with this, countermeasures were introduced during the COVID‐19 health crisis, such as wearing face masks, practising social distancing, and enhancing disinfection protocols, including the mandatory application of chemical disinfectants in both sanitary and non‐sanitary BEs (ISS 2020).

**FIGURE 2:**
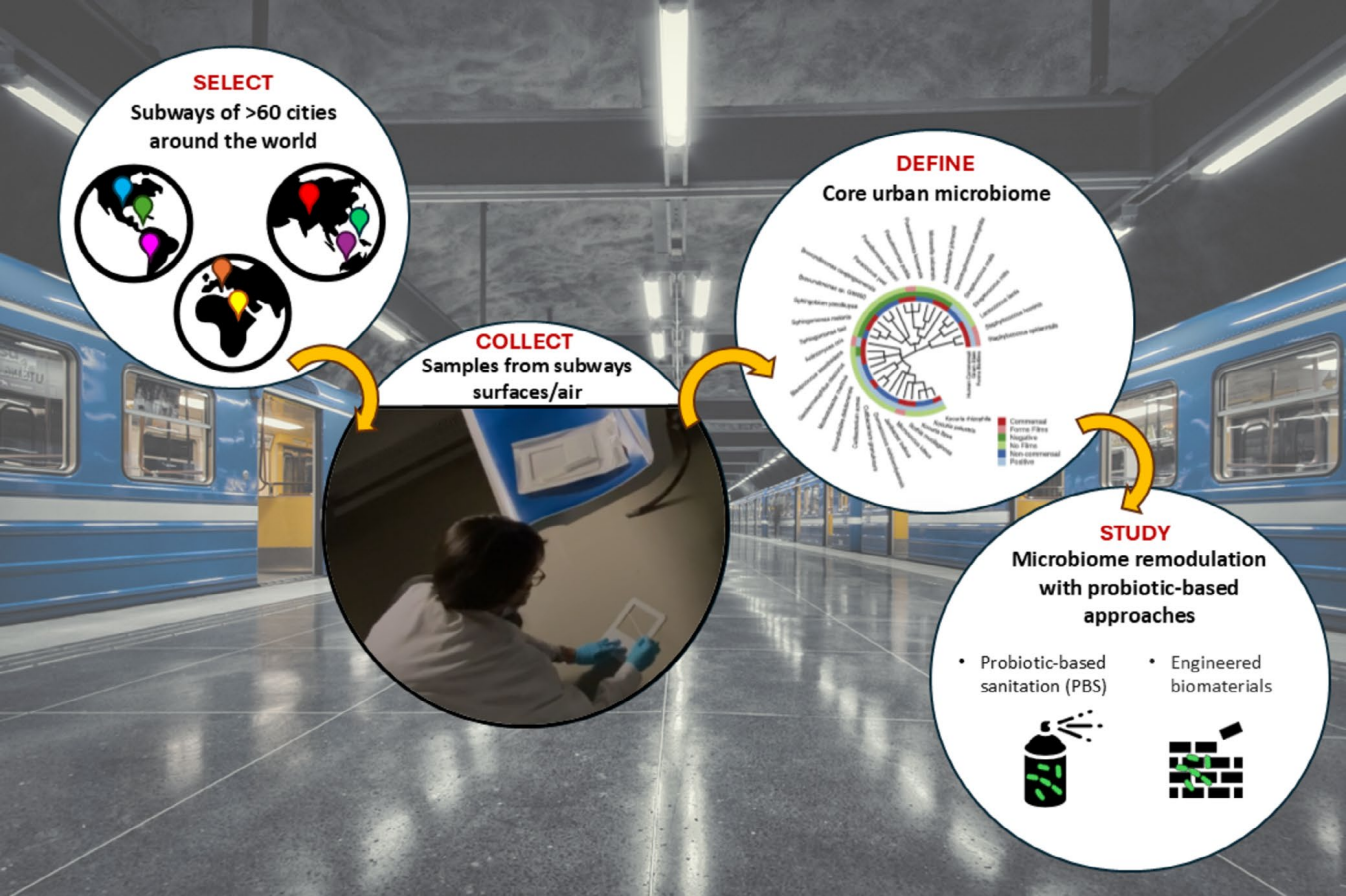
Profiling the subway microbiome by metagenomics. Deep sequencing was used to define the core urban microbiome (Danko et al. 2021) and to assess the impact of PBS on the subway microbiome (D'Accolti et al. 2023b).

Advances in metagenomics techniques have recently enabled culture‐independent analysis of the transport BE microbiome, providing taxonomic profiles, functional annotations, and monitoring of microbial AMR features (Afshinnekoo et al. 2015; Fresia et al. 2019). These data critically contributed to revealing hidden microbial reservoirs useful to track microbial transmission pathways on a global scale (Figure 2) (Zhu et al. 2017). Several studies carried out in the last decade have allowed for the characterisation of the microbiome of mass transport BE in subway settings, providing a detailed map of its composition and AMR traits across various regions worldwide (Hernández et al. 2020; Klimenko et al. 2020; Vargas‐Robles et al. 2020; Danko et al. 2021; Grydaki et al. 2021; D'Accolti et al. 2023b).

#### 1.2.1 Bacteria

Overall, the most common bacterial taxa identified in subway BE included *Staphylococcus*, *Acinetobacter*, *Propionibacterium*, *Corynebacterium*, *Micrococcus*, *Streptococcus*, and *Kocuria* genera, all of which are typical components of the human skin microbiome (Grice et al. 2008; Byrd et al. 2018; Winand et al. 2020). Some pathogenic microorganisms were also detected, such as *Helicobacter pylori* , *Acinetobacter* spp. (Kang et al. 2018), and opportunistic pathogens like *Propionibacterium acnes* , *Staphylococcus epidermidis* , and members of the genera *Pseudonocardia* and *Nesterenkonia* (Gohli et al. 2019). The majority of studies relied on 16S rRNA sequencing for microbial identification, which is not suitable for species‐level detection and cannot be used to analyse the fungal component of the subway microbiome (Winand et al. 2020; Runzheimer et al. 2024).

One of the most extensive contributions towards profiling the subway microbiome was provided by the International Metagenomics and Metadesign of Subways and Urban Biomes (MetaSUB) consortium, launched in 2015, which involved the sequencing of nearly 5000 samples derived from 60 cities worldwide (MetaSUB International Consortium 2016; Danko et al. 2021). These analyses allowed for the obtaining of a detailed atlas of the subway microbiome, including over 4000 microbial species of bacteria, archaea, and viruses, and confirmed the role of urban transit systems as a hub for microbial transmission among billions of urban residents. Of note, this study is not yet fully exhaustive, as it allowed the identification of around 80% of the sample taxa and AMR markers, but additional unique taxa and genes continue to emerge (Danko et al. 2021). Despite the geographical differences that were observed (particularly in AMR features), the study revealed a ‘core’ urban microbiome shared among cities (Danko et al. 2021), which is an important guide for future comparative studies, providing an essential reference for future comparative studies. *Proteobacteria*, *Actinobacteria* and *Firmicutes* were the three most abundant bacterial phyla detected in cities worldwide, based on the number of species observed. More specifically, over 4200 known species of urban microbes were identified, with a consistent panel of 31 species detected in all city samples (> 97% prevalence), comprising genera such as *Staphylococcus*, *Streptococcus*, *Pseudomonas*, *Brevundimonas*, *Sphingomonas* and *Kokuria* (Danko et al. 2021). The MetaSUB findings were in line with previous studies that suggested a decrease in taxonomic diversity with an increase in latitude (O'Hara et al. 2017). Each degree of distance from the equator was estimated to cause an average loss of 6.97 species in samples (Danko et al. 2021). In comparison to other areas, the Middle East and Oceania samples exhibited a greater proportion of *Firmicutes*. Despite this, functional pathways remained consistent across continents, with only minor differences in high‐level categories. Also, AMR classes varied by continent and were more consistent in taxonomically similar samples (Danko et al. 2021).

Recent studies also provided data about the subway air bacteriome, showing the prevalence of bacteria of environmental origin (such as *Acinetobacter*, *Brevundimonas*, *Lysinibacillus*, and *Clostridiodes*), accompanied by species deriving from human sources (*Flaviflexus* and *Staphylococcus*) (Sharma et al. 2024).

Other recent studies provided similar data by examining the microbiome of large railway stations that are used as hubs for various transports, thereby enabling microbial exchange across cities on a larger scale (Grydaki et al. 2021; Yan et al. 2025). The collected data evidenced the presence of microbes of both environmental (wastewater/sludge, soil, and plants) and human origin (gut, mouth, and skin). Seasonal variations in microbial diversity were observed in the study, with more α‐diversity in winter and less in spring (Yan et al. 2025). Moreover, surface samples showed a higher α‐diversity than air samples, although it was highly variable across seasons and locations (Yan et al. 2025).

Regarding water transportation systems, including ferries, boats, and cruises, the research is still limited. The microbial diversity on boats, beyond ballast tanks, has yet to be fully investigated. Although ballast water is widely recognised as a way to disperse non‐native microorganisms, there is a lack of awareness about the microbial diversity in other parts of the boat (Lymperopoulou and Dobbs 2017). Ships are complex, highly trafficked environments that have living and sleeping quarters, shared water, meals, and interconnected ventilation and sewage systems. Ships can become potential hotspots for disease outbreaks and pathogen spread due to these conditions (Prado et al. 2023). A recent investigation, carried out on a Brazilian Antarctic expedition by shotgun metagenomic analysis, revealed that bacteria, eukaryotes, viruses, and archaea account for 83.7%, 16.2%, 0.04%, and 0.002%, respectively, of the total microbiome. *Proteobacteria* was the most abundant bacterial phylum, followed by *Firmicutes*, *Actinobacteria*, and *Bacteroidetes*. Interestingly, at the beginning and end of the expedition (with few passengers on the ship), environmental bacteria were prevalent, including *Pseudomonas* spp. and *Massilia* spp., whereas during the expedition, human microbes were the most abundant, including *Cutibacterium* and Staphylococcus spp. (Prado et al. 2023). Table 1 summarises the main phyla and genera that emerged from the studies on the subway microbiome.

**TABLE 1:** The core urban microbiome emerged from studies in subway BE.

Phylum	Genus/Species	Area	BE type (sampled matrix)	Microbial detection techniques	References
Firmicutes Proteobacteria Actinobacteria	Genus: *Acinetobacter*, *Brevundimonas*, *Lysinibacillus*, *Clostridiodes*, *Flaviflexus*, *Staphylococcus*	Seul (South Korea)	Air (PM10)	NGS (16S rRNA)	Sharma et al. (2024)
Firmicutes Proteobacteria Actinobacteria	Genus: *Staphylococcus*, *Streptococcus*, *Pseudomonas*, *Brevundimonas*, *Sphingomonas*, *Cutibacterium*, *Kokuria*; Species: *L. lactis* , *S. maltophilia* , *A. johnsonii* , *M. osloensis* , *P. yeei* , *A. oris* , *B. saxobsidens* , *G. obscurus* , *M. marinus* , *N. dokodonensis*, *D. nishinomiyaensis* , *J. indicus* , *M. luteus* , *R. mucilaginosa*	60 cities	Mass transport surfaces (railings, benches, ticket kiosks)	WGS	Danko et al. (2021)
Firmicutes Proteobacteria Actinobacteria Bacteroidota Cyanobacteria	Genus: *Burkholderia‐Caballeronia‐Parabulkolderia*, *Massili*, *Deinococcus*, *Chloroplast*, *Sphingomonas*, *Staphylococcus*, *Friedmanniella*, *Paracoccus*, *Methylobacterium*, *Novosphingobium*, *Rhodanobacter*, *Nesterenkonia*	Milan (Italy)	Subway (floor, seats, handrails, doors, air filters)	NGS (16S rRNA, V3 region)	D'Accolti et al. (2023)
Firmicutes Proteobacteria Actinobacteria	Genus: *Paracoccus*, *Sphingomonas*, *Kokuria*, *Acinetobacter*, *Staphylococcus*	Athens (Greece)	Subway (bioaerosol)	NGS (16S rRNA and ITS)	Grydaki et al. (2021)
Firmicutes Proteobacteria Actinobacteria	Genus: *Stenotrophomonas*, *Pseudomonas*, *Dietzia*, *Brevundimonas*, *Intrasporangiaceae*, *Arsenicicoccus*, *Comamonadaceae*, *Staphylococcus*, *Rhodococcus*, *Erwinia*	Moskow (Russia)	Subway (railings near escalator, bench, information stand, wall, floor)	NGS (16S rRNA, V4 region)	Klimenko et al. (2020)
Firmicutes Proteobacteria Actinobacteria	Genus: *Acinetobacter*, *Corynebacterium*, *Streptococcus*, *Staphylococcus*, *Propionibacterium*, *Kokuria*, *Pseudomon*, *Micrococcus*	Mexico City (Mexico)	Subway (station turnstiles, stair and escalator handrails, platform floor, train poles, seats)	NGS (16S rRNA, V3‐V4 region)	Vargas‐Robles et al. (2020)
Firmicutes Proteobacteria Actinobacteria	Genus: *Corynebacterium*, *Propionibacterium*, *Streptococcus*, *Staphylococcus*	Mexico City (Mexico)	Subway (station turnstiles, vertical handrails)	NGS (16S rRNA, V3‐V4 region)	Hernández et al. (2020)
Actinobacteria Proteobacteria Firmicutes Bacteroidetes Cyanobacteria	Genus: *Micrococcus*, *Staphylococcus*, *Rubrobacter*, *Sphingomonas*, *Streptococcus*, *Hymenobacter*, *Corynebacterium*	Oslo (Norway)	Subway (ticket kiosks, railings, benches, air samples, air filters)	NGS (16S rRNA, V3‐V4 region)	Gohli et al. (2019)
Firmicutes Proteobacteria Actinobacteria	*Propionibacterium*, *Staphylococcus*; Species: *M. luteus* , *A. baumannii* , *E. coli*	Hong Kong	Subway (human hands after contact with handrail)	WGS	Kang et al. (2018)
Proteobacteria	Genus: *Methylobacterium*	Barcelona (Spain)	Subways (bioaerosol inside trains, platforms, lobbies)	NGS (16S rRNA, V6‐V8 region)	Triadó‐Margarit et al. (2017)
Firmicutes Proteobacteria Actinobacteria	Genus: *Corynebacterium*, *Propionibacterium*, *Streptococcus*, *Staphylococcus*	Boston (USA)	Subway (seats, poles, grips, ticket kiosks)	NGS (16S rRNA, V4 region)	Hsu et al. (2016)
Firmicutes Proteobacteria Actinobacteria	Genus: *Acinetobacter*, Species: *P. stutzeri* , *S. maltophilia* , *E. cloacae* , *L. sphaericus* , *E. casseliflavu*, *B. diminuta* , *A. lwoffii* , *B. cereus*	New York City (USA)	Subway (station turnstiles, handrails, platform floor, train poles, seats, doors, ticket kiosks)	WGS	Afshinnekoo et al. (2015)
Firmicutes Proteobacteria Actinobacteria	Genus: *Corynebacterium*, *Propionibacterium*, *Micrococcus*, *Staphylococcus*, *Enhydrobacter*	Hong Kong	Subway (bioaerosol)	NGS (16S rRNA, V4 region)	Leung et al. (2014)

##### 1.2.1.1 Bacterial Pathogens Detected in Mass Transportation

Several human pathogens, including those that cause HAIs, were persistently detected in transport BEs, which could pose a threat to human health (Mulani et al. 2019; De Oliveira et al. 2020; Denissen et al. 2022). HAI‐associated pathogens were collectively defined by WHO as the “ESKAPE” group and include *Escherichia coli* , *Staphylococcus* spp. (including *Staphylococcus. aureus* ), *Klebsiella pneumoniae* , *Acinetobacter baumannii* , *Pseudomonas aeruginosa* , *Enterococcus faecium* , and *Enterobacter* spp. (Miller et al. 2024; Denissen et al. 2022; Ly et al. 2024). The highly virulent and drug‐resistant characteristics of all of them make it difficult to treat infections when contracted (De Oliveira et al. 2020; Denissen et al. 2022). The main pathogens detected in mass transport systems are summarised in Table 2.

**TABLE 2:** Main pathogens detected in mass transport.

Pathogen	Be type	Resistance	Prevalence	References
*Enterococcus faecium/faecalis*	Subways (New York)	Tetracyclines, Beta‐lactams Aminoglycosides	27% on buses	Afshinnekoo et al. (2015); Lopes et al. (2024)
*Staphylococcus aureus*	Buses and trains (Portugal)	Meticillin, Vancomicin	16.1% on buses, 8.9% on trains, 37.1% among students	Lutz et al. (2014); Afshinnekoo et al. (2015); Mendes et al. (2015); Smelikova et al. (2025)
*Klebsiella pneumoniae*	Subways (China)	Carbapenems	1.8%	Cao et al. (2020)
*Enterobacter*	Shared bicycles (China)	—	0.1%–13.8%	Gu et al. (2020)
*Escherichia coli*	Buses and subways (China)	Ampicillin, Cefotaxime, Fosfomycin, Gentamicin, Colistin	3%	Shen et al. (2018)

Staphylococci are significant human pathogens, causing severe diseases including sepsis. They are transmitted via direct or indirect contact, since they can survive in dry conditions and persist for long periods on frequently touched surfaces. Nevertheless, the environmental monitoring of these pathogens in non‐sanitary BEs is very limited, and the details of their AMR features in these settings remain unclear. Indeed, *Staphylococcus* is the most frequently reported genus in mass transport BE, being prevalent on buses and subways, where seats and seat rails represent the most contaminated surfaces. Among them, methicillin‐resistant *S. aureus* (MRSA) was detected on buses serving both community and hospital routes, consistently including both community‐associated SCCmec type IV and healthcare‐associated SCCmec type II (Lutz et al. 2014). Of note, 65% of MRSA isolates also exhibited an MDR phenotype (Lutz et al. 2014; Afshinnekoo et al. 2015; Mendes et al. 2015; Lin et al. 2017; Angbuhang et al. 2018; Medveďová and Györiová 2019). MRSA, as well as *S. aureus* species that are naturally associated with skin, were also found on subways (Afshinnekoo et al. 2015). Other studies reported an MRSA prevalence of 16.1% on buses and 8.9% on trains (Mendes et al. 2015). Among medical students, 37.1% carried *S. aureus* , including the EMRSA‐15 lineage (ST22‐SCCmecIVh), a common hospital‐associated MRSA strain, which was found in both transport BE and students (Mendes et al. 2015). More recently, despite the extensive disinfection performed during the COVID‐19 pandemic, both trains and ground transports (buses and trams) were found contaminated by vancomycin‐resistant *S. aureus* strains (Smelikova et al. 2025).

The carbapenem‐resistant *Enterobacteriaceae* (CRE) family is also included in the ESKAPE group due to their virulence and AMR. They produce different types of carbapenemases, enzymes capable of hydrolysing a wide range of β‐lactam antibiotics, including carbapenems. The New Delhi metallo‐β‐lactamase (*bla*_*NDM*_) carbapenemase is the most frequently harboured enzyme by *Enterobacteriaceae* in hospital settings (Khan et al. 2017). The two species *Escherichia coli* and *Klebsiella pneumoniae* are the most common hosts of *bla*_*NDM*_ (Cao et al. 2020). *E. coli* is a major cause of HAIs, including urinary tract infections (UTIs), bloodstream infections, and sepsis. The strains isolated from the hospitalised patients are almost always MDR (Denissen et al. 2022). However, MDR strains have also been detected in the mass transport BE, including strains resistant to ampicillin, cefotaxime, fosfomycin, gentamicin, and even *mcr‐1* driven colistin in 3% of isolates (Shen et al. 2018). Also, *K. pneumoniae* was identified in subways, although it was less common than *E. coli* . It was particularly found on high‐touch surfaces, where it was found to be resistant to carbapenems in 1.8% of collected samples (Cao et al. 2020).

Last, both *Enterococcus* and *Enterobacter* species were detected in mass transportation (Shen et al. 2018; Ly et al. 2024). Among Enterococci, *E. faecium* and *E. faecalis* are usually MDR and vancomycin‐resistant (VRE) in the hospital environment, where they are commonly linked to opportunistic infections and hospital outbreaks. Hospital isolates are also capable of tolerating various stressors (such as starvation and disinfectants) and can cause endocarditis, UTIs, bloodstream infections, post‐surgical wounds, and sepsis (Chilambi et al. 2020; Zhou et al. 2020). *E. faecium* was found in New York subways (Afshinnekoo et al. 2015) and on highly touched surfaces of public buses in Lisbon (Lopes et al. 2024). In particular, the recent study by Lopes and colleagues highlights the widespread presence of clinically relevant and drug‐resistant *Enterococcus* species in non‐clinical settings, such as public buses and passengers' hands. The prevalence of *E. faecium* and *E. faecalis* on bus surfaces was 27% and 46%, respectively (Lopes et al. 2024). A significant presence of MDR *E. faecalis* (up to 13.8% of collected samples) was also found on shared bicycles in China (Gu et al. 2020). The *Enterobacter* genus, belonging to the *Enterobacteriaceae* family, includes some species primarily associated with HAIs, such as *Enterobacter cloacae* and *Enterobacter aerogenes* . *Enterobacter* infections are associated with an extensive range of clinical manifestations and have become increasingly resistant to many antibiotics, including carbapenems (CRE). *Enterobacter cloacae* , the most clinically relevant species, was detected with high abundance in the mass transportation environment (Afshinnekoo et al. 2015).

#### 1.2.2 Fungi

Fewer studies focused on the mycobiome composition in transport areas, compared to bacteriome profiling. However, it was reported that airborne fungi were present in the Seoul subway, with samples taken from both workers' areas (station office, bedroom, ticket office, and driver's seat) and passengers' areas (station, passenger carriage, and platform). The fungal genera detected with a relative abundance ≥ 5% included the *Penicillium*, *Cladosporium*, *Chrysosporium* and *Aspergillus* genera. *Penicillium* and *Cladosporium* accounted for > 60% of the total airborne fungi (Kim et al. 2011). Similarly, studies performed in New York (Robertson et al. 2013) and Athens (Grydaki et al. 2021) showed the prevalence of *Ascomycota* and *Basidiomycota*. In accordance with previous indoor and outdoor bioaerosol studies (Hoisington et al. 2014; Shin et al. 2015), *Dothideomycetes* and *Agaricomycetes* were the prevalent fungal classes. *Cladosporium* was the most abundant genus, often dominating BE air mycobiomes (Fröhlich‐Nowoisky et al. 2012). *Cladosporium* was also reported as the dominant fungus in Athens' outdoor air (Pyrri and Kapsanaki‐Gotsi 2015; Richardson et al. 2019). *Mycosphaerella* (including species that infect plant leaves) was the second most abundant fungal genus in Athens subways. Both *Cladosporium and Mycosphaerella* belong to the *Capnodiales* order, and they have also been identified in the New York subway (Robertson et al. 2013). Other common fungal genera included *Penicillium*, *Aspergillus* and *Alternaria*, which are frequently found in indoor environments (Nevalainen et al. 2015).

#### 1.2.3 Viruses

Most research on the BE microbiome has primarily focused on bacteria and fungi, often neglecting viruses, which have been referred to as “the forgotten siblings of the microbiome family” (Williams 2013). In contrast, it has been found that indoor air has as many viruses as bacteria (Prussin and Marr 2015). Although the reference database and bioinformatic pipelines are limited, metagenomic approaches have allowed for the simultaneous identification of multiple viruses, highlighting that the urban microbiome harbours a significant, unexplored viral diversity that has not been seen in other environments. Specifically, the MetaSUB metagenome‐assembled genomes (MAGs) identified 11,614 viral species, but 94.1% had no match to any viral sequence in the Integrated Microbial Genome and Viral Database (IMG/VR) (Paez‐Espino et al. 2019). This results in 10,928 viruses that do not correspond to known species (Danko et al. 2021). The analysis of predicted viral hosts aligned with the taxonomic profiles, as over 40% of species in the core microbiome had predicted viral‐host interactions. Many of the viral MAGs were found in multiple locations, including South America, North America, and Africa. Viral MAGs in Japan often corresponded to those in Europe and North America (Danko et al. 2021). The study by Prado and colleagues employed a shotgun metagenomic approach, capturing both DNA and RNA, representing so far the most comprehensive evaluation of indoor virome in transportation facilities (Prado et al. 2023). Through de novo assembly, the identification of ten viral families was made: *Autographiviridae*, *Chrysoviridae*, *Genomoviridae*, *Herelleviridae*, *Myoviridae*, *Partitiviridae*, *Podoviridae*, *Potyviridae*, *Siphoviridae* and *Virgaviridae* (Prado et al. 2023). RNA viruses were resulted more prevalent than DNA viruses, and a number of bacteriophage families were identified. They included *Pahexavirus* phages (infecting *Propionibacterium*), *Actinomyces_virus_Av1* phage (a *Podoviridae* virus infecting *Actinomyces*, frequently present in the human mouth), and *Siphoviridae* (phages infecting *Staphylococcus*). In addition, several insect‐infecting viruses from the *Nudiviridae* and *Polydnaviriformidae* families were identified (Prado et al. 2023).

### 1.3 The Control of Bioburden in Mass Transport BE

Based on the persistent microbial bioburden detected in transport areas, surface disinfection and air purification appear to be crucial tools to prevent pathogen transmission in these areas. Chemical disinfection has been the most frequently adopted method for many years, but it has some significant limitations. Thus, alternative sanitising methods have been proposed, prioritising sustainable and green technology, including the use of UV‐C light, disinfectant fumigation, plasma air sterilisation, antimicrobial surfaces (Ly et al. 2024), and PBS (D'Accolti et al. 2022, 2024; Neidhöfer et al. 2023; Denkel et al. 2024) (Table 3).

**TABLE 3:** Main infection control methods in mass transport systems.

Method	Mechanism of action	Advantages	Limitations
Chemical disinfection	Membrane disruption Macromolecule dysfunction Metabolic inhibition	Low costs Rapid effect Ease of application Well established use	High environmental impact Harmful to humans Temporary action Favour the selection of MDR pathogens
UV‐C Light Sterilisation	DNA damage mediated by the generation of reactive oxygen species (ROS)	Low costs Combinable with disinfection	Harmful to humans Cause material damage Not effective on shadow areas
Hydrogen peroxide/peracetic acid fumigation	Oxidation and irreversible damage of microbial compounds	Effective also against bacterial spores	Effectiveness dependent on the type of material, microorganism, fumigation device, and technology
Plasma air sterilisation	Combined action of charged particles, reactive species, UV‐C radiation, heating	Well established use (food industry and medical field)	Need of extensive validation in the context of public transport
Antimicrobial surfaces	Cu^2+^ ions induce membrane damage and ROS production, and reduce microbial adhesion	Cost‐effective	Not suitable for all types of surfaces Scarcely sustainable in terms of costs

#### 1.3.1 Chemical Disinfection

Conventional chemical disinfection has represented the mostly used approach to control bioburden for decades, in both sanitary and community environments, and was massively increased during the COVID‐19 pandemic to manage the emergence of SARS‐CoV‐2 transmission (CDC 2020, 2021; ISS 2020). Disinfectants are rapidly effective and easily applied, but they also have undesirable side effects (NPSA 2007). Specifically, chemical disinfectants have a significant impact on the environment, resulting in pollution of soil and water ecosystems (Nabi et al. 2020; Zhang et al. 2020). Furthermore, more than half of disinfectant‐treated surfaces remain inadequately decontaminated (Carling et al. 2008), and many microbes persist even after treatment (Kramer et al. 2006; Goodman et al. 2008). The disinfectants' effects are short‐lived, and recontamination takes place quickly on treated surfaces, reconstituting the original bioburden levels in 30–120 min (Rutala and Weber 2014; D'Accolti, Soffritti, Bonfante, et al. 2021). Last, disinfectants can favour the selection of microbes that develop tolerance and/or resistance to antimicrobials (Kampf 2018). For example, chlorhexidine has the ability to promote resistance to a wide range of drugs, including colistin, ceftazidime, imipenem, and tetracycline (Kampf 2018). Similarly, benzalkonium chloride adaptations can result in resistance to antibiotics like ampicillin and cefotaxime. Consistent with this, an alarming global increase in AMR was recorded during the COVID‐19 pandemic, when massive chemical disinfection was mandatorily introduced worldwide (Clancy et al. 2020; Lai et al. 2021), highlighting the risks that these methods pose to human health.

#### 1.3.2 UV‐C Light Sterilisation

UV‐C radiation (200–280 nm wavelength) is effective since it induces DNA damage by the generation of reactive oxygen species (ROS) in microbial cells (Murphy 1975; Peak et al. 1985). This method has low operating costs (Rakib et al. 2022) and has been used in hospitals, often in combination with chemical disinfection (Guettari et al. 2021; Santos and Santos and de Castro 2021). However, disadvantages include the fact that it can be harmful to humans, it can cause damage to materials, and it cannot reach shadow areas typically found in complex surfaces of cabins and waggons (Teska et al. 2020; Ly et al. 2024).

#### 1.3.3 Disinfectant Fumigation

Fumigation involves the use of chemical antimicrobial solutions, such as hydrogen peroxide or peracetic acid, to disinfect a specific area. In mass transport settings, fumigation showed effectiveness in decontaminating buses and was also active against bacterial spores, which are known to be resistant to most disinfectants (Leggett et al. 2015). The drawbacks are that it can only be used in the absence of human presence, and it can cause damage to certain materials.

#### 1.3.4 Plasma Air Sterilisation

Plasma disinfection is the result of the combined action of charged particles (ions, electrons), reactive species (ozone, ROS), UV‐C/Vacuum‐UV (VUV) radiation, and heating (Laroussi 2005; Gallagher et al. 2007; Scholtz et al. 2015). So far, its application has been limited to the food industry and the medical area (Bernhardt et al. 2019; Deng et al. 2020; Borges et al. 2021; Hong et al. 2023), but a first plasma‐related method, based on needle‐point bipolar ionisation, has been recently shown to decrease environmental bioaerosols in tramway settings (Baselga et al. 2023). Also in this case, the main disadvantages are that it cannot be used in the presence of humans and it can cause damage to specific materials.

#### 1.3.5 Antimicrobial Surfaces

Self‐disinfecting surfaces, containing antimicrobial compounds such as copper, iron, and silver, have also been proposed to reduce the microbial load on frequently touched surfaces (Lansdown 2006; Noyce et al. 2006; Casey et al. 2010). However, they are not appropriate for every surface type and are scarcely sustainable in terms of costs (Dancer 2014). Copper's antibacterial properties are caused by the release of Cu^2+^ ions, resulting in cell membrane rupture, leading to the loss of membrane potential and the depletion of cytoplasmic substances (Grass et al. 2011). In addition, Cu^2+^ ions generate ROS products, which can cause DNA damage (Hong et al. 2019). Although copper can be expensive as a raw material, integrating it as metal nanoparticles in a polymer matrix can be a cost‐effective alternative, taking advantage of its antibacterial properties. This approach makes it easier for copper‐based materials to be widely used (Tamayo et al. 2016). Other types of studied antimicrobial surfaces include anti‐biofouling surfaces to reduce microbial adhesion, biocidal nanocomposites able to kill bacteria, and nanostructured surfaces that destroy bacteria through physical mechanisms (Cassidy et al. 2020; Mahanta et al. 2021; Linklater et al. 2021). Some antimicrobial materials have already been tested in mass transportation, leading to variable results. For example, antimicrobial photodynamic coatings showed a significant 22.6% reduction of bacterial counts (Kalb et al. 2022), whereas photocatalyst‐coated and uncoated hand‐contact surfaces did not provide any statistically significant drop in microbial burden (Eicker and Salomon 2021).

#### 1.3.6 PBS

PBS is a unique approach among the recent proposed procedures, as it involves the addition of beneficial bacteria (probiotics) instead of eliminating all microbial species in the treated environment. Probiotics are defined by the WHO as ‘live microorganisms that, when given in sufficient amounts, provide a health benefit to the host’, and they are essentially used in living organisms, such as humans (Hill et al. 2014). They are widely regarded as safe and have been shown effective for the treatment of a variety of human health conditions, including gut and urinary diseases, oral pathologies (gingivitis and periodontitis), antibiotic‐resistant skin infections, and allergic disorders (National Institute of Health 2020). Their action largely relies on their ability to outcompete pathogens for nutrients and space via competitive exclusion, production of antimicrobial compounds, and ability to shape the microbial community through quorum sensing. Many different species of probiotics can be used, depending on the type of action desired, as both the mechanism of action and the induced effects are highly species‐ and strain‐specific.

The comprehension of human and environmental microbiome ecosystems led to the recognition that a bidirectional hygiene (“bygiene”) approach could be more useful compared to disinfection since it leads to pathogen reduction via the counterbalance exerted by beneficial microbes (Al‐Ghalith and Knights 2015). This can preserve microbial diversity (CDC 2020) and provide a microbial community that can prevent colonisation by pathogens and AMR spread. In healthcare settings, PBS has been shown to yield promising results. The majority of studies have focused on a system that utilises an eco‐friendly detergent and probiotics from the *Bacillus* genus. These spore‐forming probiotics are ubiquitously found in the environment and classified as non‐pathogenic (EFSA Panel on Biological Hazards (BIOHAZ) et al. 2022). Bacterial spores are particularly convenient for sanitation purposes since they can survive in the concentrated detergent and resist a wide range of temperatures, concentrations of ionic and anionic compounds, and pH levels. After appropriate dilution in water, *Bacillus* spores can germinate and colonise the surfaces where they are spread, outcompeting the resident microbes and preventing pathogens' colonisation (Gottel et al. 2024). In particular, the system tested in sanitary environments (named PCHS, Probiotic Cleaning Hygiene System) included the species *Bacillus subtilis* , *Bacillus velezensis* and *Priestia megaterium*.

PCHS implementation has been shown to result in a permanent decrease of pathogens, as well as in AMR and in associated HAIs, in comparison to disinfectants (Mazzacane 2014; Vandini et al. 2014; Caselli and Mazzacane 2016; Caselli et al. 2018, 2019; D'Accolti et al. 2018, 2023a, 2023b, 2024; D'Accolti, Soffritti, Mazzacane, et al. 2019; D'Accolti, Soffritti, Bonfante, et al. 2021; La Fauci et al. 2018; Comar et al. 2019; Klassert et al. 2022; Soffritti et al. 2022; Leistner et al. 2023; Neidhöfer et al. 2023; Ramos and Frantz 2023; Gottel et al. 2024). The most extensive trial of PCHS effectiveness consisted of a multicentre study lasting 18 months, performed in six Italian public hospitals receiving PBS in place of conventional chemical‐based disinfection (Caselli et al. 2018). Throughout the study period the hospital surface bioburden, AMR, and HAI incidence were monitored, resulting in the analysis of 24,875 environmental samples and 11,842 patients. Collected data showed that PCHS use was associated with a stable decrease of surface ESKAPE pathogens (−83%; range 70%–96.3%) and a −99% drop of pathogens' AMR genes, compared to what was obtained with chemical disinfection (*p* < 0.0001) (Caselli et al. 2018). Consistent with the significant reduction of bioburden and AMR, PCHS induced a significant −52% decrease of HAI incidence, from a global 4.8% (284 patients with HAI over 5930 total patients) to 2.3% (128 patients with HAI over 5531 total patients) (*p* < 0.0001). Other studies were subsequently performed in other hospital settings in Italy, Germany, Russia, South Africa, and the Arabian Emirates, confirming the ability of similar PBS to control pathogenic bioburden and AMR (reviewed by D'Accolti et al. 2024; Denkel et al. 2024). Based on collected data, the Robert Koch Institute Commission for Hospital Hygiene and Infection Prevention included PBS as a sustainable way to provide a long‐term stable microbiome without favouring the development of cross‐resistance to antibiotics in its recently released recommendations (Koch‐Institut 2022). Based on these premises, the use of probiotics as antimicrobial agents has recently been proposed also for non‐sanitary BEs, including mass transportation (D'Accolti et al. 2023b; Timmis et al. 2025).

### 1.4 Probiotic‐Based Approaches for Infectious Control in Mass Transport

**FIGURE 3:**
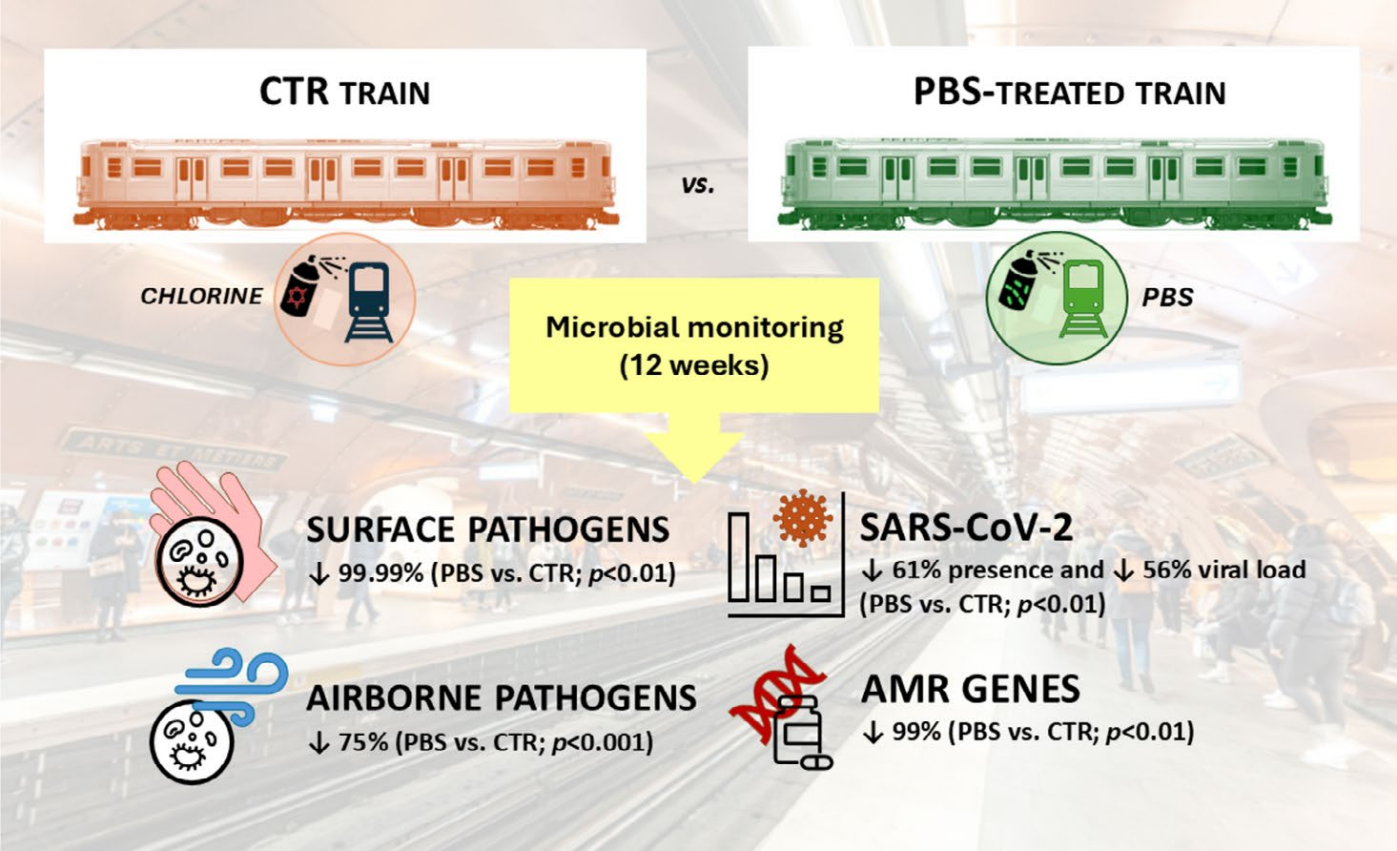
Probiotic‐based sanitation (PBS) in subways. The design and main data collected in a single‐centre study are summarised (D'Accolti et al. 2023b). The percentage decrease of surface and air pathogens, SARS‐CoV‐2 presence, and antibiotic‐resistance genes in PBS‐treated vs. CTR train are indicated.

The eco‐friendly PCHS system, which contains *Bacillus* probiotics, was the main method used for probiotic sanitation studies in non‐sanitary community settings (Caselli et al. 2019). Recent research was undertaken to examine the applicability and effectiveness of this type of PBS in mass transportation, based on previous results obtained in the sanitary environment (Figure 3). Specifically, the study was carried out as a pre‐post and case–control study during the COVID‐19 emergency in an Italian subway to evaluate the effects of PBS compared to conventional chlorine‐based chemical disinfection. The PBS detergent contained 10^7^/mL spores from the following three species of *Bacillus: B* *. subtilis* , *B. velezensis* (formerly classified as *Bacillus pumilus* ), and *P. megaterium* (formerly named *B. megaterium* ) (D'Accolti et al. 2018).

PBS was used for both surface and air cleaning (through wipes and nebulization, respectively). PBS was used to substitute for chemical disinfection in the treated train, while alcohol and chlorine were used to disinfect surfaces and air in the control train. Surfaces and air were monitored throughout the entire two‐month study period, to profile the microbiome of the trains. The analysis was conducted using both culture‐dependent (CFU counts) and molecular methods (quantitative real‐time PCR, microarray, and NGS). Bacterial, fungal, and viral pathogens of human origin (including SARS‐CoV‐2) were measured, along with the level and type of bacterial AMR. In addition, the composition of the whole microbiome was profiled by 16S rRNA NGS. The results demonstrated that the subway environment was home to multiple human pathogens, which confirmed their persisting presence in high‐traffic BEs. PBS usage, when compared to chemical disinfection, resulted in a significant elimination of human pathogens from the train surface and air, resulting in the virtual disappearance of pathogens at the end of the study period (−99.99%, *p* < 0.01) (D'Accolti et al. 2023b). Notably, several genes expressing drug resistance were detected in trains at baseline. They included genes conferring resistance against beta‐lactams (*ACT‐5/7* group, *OXA‐2* and *OXA‐23* groups), erythromycin and streptogramin (*ermA*, *ermC* and *msrA*), and methicillin (*mecA*), confirming the spread of resistant bacteria, including virulent MRSA, outside of the hospital environment (D'Accolti et al. 2023b). PBS induced an 85% reduction of all the originally detected AMR genes, compared to chemical disinfection, which confirmed the results obtained in hospitals (D'Accolti et al. 2023b). Both trains were found to have SARS‐CoV‐2 at baseline, a confirmation of the virus's spread in the transportation environment during the COVID‐19 pandemic. PBS also reduced by 80% the presence of SARS‐CoV‐2 in the subway BE, compared to chemical disinfection, supporting its usefulness in stably reducing the pathogen bioburden, including viruses. These results were in accordance with previous demonstrations of PBS antiviral activity in vitro and in situ in the sanitary settings (D'Accolti et al. 2020, 2023a; D'Accolti, Soffritti, Bonfante, et al. 2021; Soffritti et al. 2022). Interestingly, the whole composition of the subway microbiome was not significantly affected by PBS, while the human pathogenic components (representing less than 10% of the total microbiome) were significantly decreased. In contrast, PBS led to a significant change in the microbiome profile in hospitals (Caselli et al. 2018). This difference may be related to the diverse composition of the train microbiome compared to the hospital one. Indeed, while the microbiome of the hospital environment is predominantly of human origin, the train microbiome appears to be predominantly influenced by environmental species, the percentage of which is less affected by the addition of *Bacillus*. In contrast, human pathogens, which are characterised by their high nutrient requirements, could be more effectively tackled and inhibited by PBS due to the mechanism of competitive exclusion (D'Accolti et al. 2023b).

In addition to their use as sanitisers, the direct incorporation of *Bacillus* probiotics into material engineering is an intriguing potential application (González et al. 2019). Probiotics have in fact been proposed not only for sanitation purposes, and early‐stage studies have considered the potential of adding probiotics directly to materials. In this regard, recent data suggest the possibility of including *Bacillus* spores directly into materials to confer their antimicrobial properties (González et al. 2019). Included *Bacillus* probiotics could also repair microfractures (Nguyen et al. 2019; Su et al. 2021; Nodehi et al. 2022) and induce shape changes in response to humidity variations (Birch et al. 2021). This research paves the way for the potential incorporation of probiotic spores into various materials. This would help maintain structural integrity and enhance occupant health through biocontrol mechanisms (Lax et al. 2015), with potential interesting applications in mass transport. In this regard, 3D printing may play a key role in the future of microbial biocontrol in BEs. Recent advancements in 3D printing have allowed printing materials at temperatures low enough to preserve the viability of spores and even live bacteria during the assembly process (González et al. 2019). Unlike previous methods that relied on hydrogels, these new approaches permit the printing of ceramics and hard plastics, which are the most commonly used materials in BEs, including transport spaces. Specific probiotics optimised for these printing techniques could lead to improved products and materials, allowing the design of strategies for a stable balance of the BE microbial ecology, ultimately enhancing occupant health and human well‐being (González et al. 2019). Table 4 summarises the main studies on probiotic usage in mass transport.

**TABLE 4:** Main studies on the use of probiotics in mass transport.

Probiotic‐based approach	Study type	BE type	Main outcomes	References
Probiotic‐based sanitation ( *B. subtilis* , *B. velezensis* , and *P*. *megaterium* spores)	In situ	Subways (Italy)	Significant pathogens' decrease (up to −99.99%, *p* < 0.001) including SARS‐CoV‐2 −85% reduction of AMR	D'Accolti et al. (2023)
Engineering materials using 3D printing ( *B. subtilis* spores)	In vitro	—	Engineered materials resilient to extreme stresses (desiccation, solvents, osmolarity, pH, ultraviolet light, and γ‐radiation) Spores germinate on material surfaces and can produce chemicals on demand.	González et al. (2019)
Engineering materials using multiple monolayers of *B. subtilis* spores and latex sheets	In vitro	—	Bacterial Spore‐Based Hygromorphs: engineered material able to respond to changes in relative humidity (RH) and wetting through shape change	Birch et al. (2021)
Concrete with microbial adjuvant of *Bacillus* spores ( *B. subtilis* , *B. megaterium* )	In vitro	—	Bacterial self‐healing of concrete: engineered material able to repair open micro‐cracks by CaCO_3_ precipitation	Nguyen et al. (2019); Su et al. (2021)

#### 1.4.1 Regulatory Considerations and Safety Assessments of PBS

Although PBS appears very promising for controlling bioburden and infectious risk in sanitary settings, its application in mass transportation areas is still in the early stages. While there are numerous microbial‐based cleaners that can be purchased, there is currently no regulation mandated for the probiotic microorganisms in these products (Arvanitakis et al. 2018). In Europe, microbial‐based cleaning products are only governed by regulations focused on the safety of biological agents at work (Arvanitakis et al. 2018). In the United States, the probiotic species used in commercial cleaners are classified as food‐grade with a GRAS (generally recognised as safe) label, meaning no additional regulations are required to assess their efficacy or safety (Velazquez et al. 2019). Recent studies have indicated that manufacturers have significant differences in toxicological risk assessments, hygienic practices, and quality control practices (Teasdale and Kademi 2018). Despite the increase in voluntary eco‐labelling, certification is often centred on human safety, product effectiveness, and environmental impact, without requiring specific information about the microbe consortium's identity or concentration (Iraldo et al. 2020). Manufacturers typically keep the exact identity and composition of the microbes confidential, which is why product labels generally only mention taxonomic genera (Arvanitakis et al. 2018).

Moreover, despite *Bacillus* safety being assessed in sanitary studies (Caselli et al. 2016; Bini et al. 2025), the widespread use of PBS in community BEs would significantly elevate human exposure to *Bacillus* spores and vegetative cells, suggesting the need for long‐term studies to assess any potential ecological impacts of PBS on the human microbiota and its effects on human health (Ramos and Frantz 2023).

## 2 Challenges and Future Perspectives

Overall, probiotic‐based applications appear very promising and smooth to control bioburden in high‐traffic human environments, such as mass transportation, where gradual long‐term stabilisation of the persistent microbiome can be effective in lowering the infectious risk. The biological nature of PBS and other probiotic‐based approaches is the main drawback. First, due to its microbial nature, PBS is not compatible with simultaneous and continuous disinfection with chlorine or other sporicidal disinfectants, which inactivate probiotics, preventing their effect (D'Accolti, Soffritti, Mazzacane, et al. 2019; D'Accolti et al. 2024). However, some non‐sporicidal disinfectants can be used, as demonstrated by in situ studies (Soffritti et al. 2022; D'Accolti et al. 2023a, 2023b), opening the way to eventual combined strategies. Second, due to its mechanism of action (competitive exclusion), PBS requires two to four weeks to stably modulate the microbiome of the treated BE, making it more suitable for long‐term prevention than rapid decontamination (Caselli 2017). Also, PBS action is nonspecific, impacting gradually on all kinds of potential pathogens. Therefore, it is not ideal when rapid decontamination is needed against a specific pathogen. Last, while PBS is generally suitable for non‐sanitary environments, it is not recommended for areas that require sterility, like surgical rooms. However, it has been determined to be safe for hospitalised patients with particularly fragile conditions, including elderly individuals, moderately immunocompetent individuals, and adult and newborn ICU patients (Caselli et al. 2016, 2018). Further research could explore the use of probiotic‐derived molecules, such as enzymes or bacteriocins, for applications in sterile environments or high‐risk settings.

In order to tackle the limitations of PBS in terms of specificity and rapidity of action, lytic bacteriophages have been proposed as a potentially effective strategy (D'Accolti et al. 2018, 2023a; D'Accolti, Soffritti, Lanzoni, et al. 2019; D'Accolti, Soffritti, Mazzacane, et al. 2021). Lytic phages, which are highly specific prokaryotic viruses, may kill target bacteria very rapidly (within 1 h) and in an extremely specific way, without perturbing the rest of the present bacteriome or disturbing the present probiotics (D'Accolti, Soffritti, Mazzacane, et al. 2021). The use of PBS and lytic phages has been consistently reported to be effective in abating target bacteria in a specific way (D'Accolti et al. 2018, 2023a; D'Accolti, Soffritti, Lanzoni, et al. 2019). Similarly, phage‐derived lytic enzymes (endolysins) may be added to PBS to increase the killing of a wider range of bacterial targets.

Of note, although *Bacillus* probiotics are considered safe according to FDA and EFSA requirements (Gad 2005; EFSA Panel on Biological Hazards (BIOHAZ) et al. 2022), extensive safety studies have been performed only in sanitary settings on specific *Bacillus* strains. In detail, PBS‐probiotics were scrutinised for their infectious risk and genetic stability. Active surveillance of probiotic‐associated infection was carried out in all the hospitals using PBS, showing the complete absence of PBS‐*Bacillus* infectivity or invasiveness, even in patients with a high risk of opportunistic infections (Caselli et al. 2016). In addition, the genetic content of PBS‐*Bacillus* isolates from treated hospitals was recently analysed by WGS, providing their complete virulome, resistome, and mobilome sequence. The results demonstrated the absence of genes of concern in the original strains, as well as of any newly acquired genes in all the *Bacillus* isolates, despite the continuous contact with surrounding pathogens (Bini et al. 2025). These data demonstrate that PCHS‐*Bacillus* strains exhibit high genetic stability, confirming their long‐term safety. However, further studies would be needed for different PBS formulations intended for use in human BEs.

Overall, the routinary use of probiotic cleaning products in sanitary and community settings should require quality and safety standards that need to be monitored by public authorities at the national and international levels (Denkel et al. 2024). The European Union (EU) has recently regulated probiotic‐based cleaning products, stating that microorganisms intentionally added to detergents shall have an American Type Culture Collection (ATCC) number, belong to an International Depositary Authority (IDA) collection, or have their DNA identified at the species level by 16S ribosomal DNA sequencing or whole genome sequencing (European Commission 2023). Also, studies assessing the PBS effectiveness on dry biofilms, which are often detectable on dry surfaces and very difficult to remove via conventional disinfection (Almatroudi et al. 2018), are still lacking. The use of PBS in mass transit would benefit greatly from these studies.

Finally, further important yet neglected elements to evaluate are those concerning the carbon footprint of the cleaning service. To ensure eco‐friendly procedures, the cleaning systems should also be evaluated for the CO_2_ emission per square meter of treated surface by using the global warming potential (GWP) indicator. This would provide the carbon footprint of the activity, differentiated into the various phases of the cleaning cycle (i.e., production of the components, transportation, application, waste disposal, and timing of the interventions). Studies are currently underway aimed at proving that PBS can guarantee significantly lower GWP values than those related to the use of chemical systems (D'Accolti et al. 2025). Such studies could also quantify the eventual compensatory measures (such as tree planting) needed to reduce the atmospheric CO_2_ emitted due to the cleaning procedures used.

Addressing these questions will require a range of experimental approaches, such as testing different application methods, using a diverse range of target pathogenic microbes, and studying the dynamics of the entire microbial community in response to PBS application.

In addition, innovative PBS application strategies could be constructed to work together with other sustainable hygiene techniques, such as the use of engineered biomaterials, self‐disinfecting surfaces, and specific microbial engineering for targeted pathogen control.

Overall, robust, multi‐national, multicenter randomised controlled trials (RCTs) with sufficient statistical power would be needed to confirm the impact of PBS in hospital settings (in terms of HAI incidence and MDR acquisition) and assess its potential in community environments. Evaluating the effectiveness and long‐term sustainability of these new cleaning practices is crucial for a paradigm shift to their routine use in sanitary and non‐sanitary settings.

## 3 Conclusions

In highly crowded BEs such as mass transportation, like subways, buses, and trains, probiotic‐based approaches have the potential to be an innovative and sustainable way to counteract pathogen contamination. Based on the results obtained in various indoor environments, PBS has emerged as a sustainable system capable of stably balancing the indoor microbiome, preventing recontamination and gradually reducing pathogens and their AMR. The effect of bioburden reduction countermeasures in mass transportation, along with effective detection methods, requires further research. To enhance the safety of passengers, hand hygiene and common‐sense hygiene guidelines are crucial components of the action plan. Nevertheless, the probiotic‐based approach has the potential to provide new solutions to address current and future challenges in infectious risk and AMR control. The preservation of environmental and human health can be significantly improved by harnessing the power of beneficial microorganisms.

## Author Contributions

**Irene Soffritti:** writing – original draft, investigation. **Maria D'Accolti:** investigation, validation, data curation. **Francesca Bini:** investigation, visualization, software, data curation. **Eleonora Mazziga:** investigation, visualization, data curation. **Antonella Volta:** investigation, methodology, data curation. **Matteo Bisi:** investigation, methodology, data curation. **Sante Mazzacane:** conceptualization, supervision. **Elisabetta Caselli:** conceptualization, writing – review and editing, visualization, supervision.

## Conflicts of Interest

This work did not receive any specific funding. The authors declare no conflicts of interest.

## Data Availability

Data sharing not applicable to this article as no datasets were generated or analysed during the current study.
